# Cerebellar transcranial AC stimulation produces a frequency-dependent bimodal cerebellar output pattern

**DOI:** 10.21203/rs.3.rs-5147104/v1

**Published:** 2024-11-20

**Authors:** Devry Mourra, Angela M. Cavalieri, Madison M. Casey, Mesut Sahin, Eric J. Lang

**Affiliations:** New York University, Grossman School of Medicine; New York University, Grossman School of Medicine; New York University, Grossman School of Medicine; New Jersey Institute of Technology; New York University, Grossman School of Medicine

**Keywords:** transcranial electrical stimulation, cerebellar nuclei, Purkinje cell, electric fields in neural tissue

## Abstract

Transcranial alternating current stimulation (ctACS) has the potential to be an appealing, non-invasive treatment option for psychiatric and neurological disorders. However, its potential has been limited by significant knowledge gaps in the details and mechanisms of how ctACS affects cerebellar output on single cell and population levels. We investigated this issue by making single-unit recordings of Purkinje cells (PC) and lateral cerebellar nuclear (Lat CN) cells in response to ctACS in anesthetized adult female Sprague-Dawley rats. The ctACS electrode was positioned directly on the skull above crus 1, either ipsilaterally just medial to the recording site or contralaterally. The return electrode was placed under the skin of the shoulder ipsilateral to the recorded cell. In response to ctACS at frequencies ranging from 0.5 to 80 Hz, PC and CN activity was modulated in a frequency-dependent manner. PC and CN entrainment strength increased with stimulation frequency. Moreover, a unimodal response was seen for most PCs across all frequencies, whereas most CN cells transitioned to bimodal patterns as stimulus frequency increased. The phase of the local minima CN cells, and its change with frequency, was consistent with CN cells being driven synaptically by PC activity. Furthermore, the nearer ctACS location to the recording site, the stronger the entrainment, suggesting that ctACS electrode placement could be used to target specific cerebellar output channels. In sum, the results show that transcranial stimulation of the cerebellar cortex can modulate cerebellar output, which has potential implications for its use in treating neurological and psychiatric disorders.

## Introduction

Low-intensity transcranial electrical stimulation (tES) is generating significant interest both as a clinical therapy and scientific tool because it is non-invasive, safe, and requires only simple equipment [1–4]. In particular, cerebellar transcranial electrical stimulation (ctES) has been reported to improve motor learning, cognitive, and emotional processes in normal and brain-injured individuals [2–10]. More specifically, cerebellar transcranial alternating current stimulation (ctACS), a type of tES, has been shown to enhance motor learning and function in humans [11–13]. However, ctACS’s potential is limited by the relative lack of knowledge about how it affects neuronal activity in mammalian species at the cellular and systems levels *in vivo*.

ctACS’s therapeutic effects on brain activity certainly involve a direct action on cerebellar cortical activity, but they may also involve effects on other brain regions to which the cerebellum projects, to the extent that the cerebellar nuclei (CN), the main output station of the cerebellum, are modulated. This would greatly increase ctACS’s clinical potential for treating both motor and cognitive disorders because of the widespread projections of the CN throughout much of the cerebrum via the thalamus [14], and there is evidence that this is the case. CN modulation was observed with alternating current (AC) stimulation applied directly to the surface of the cerebellum [15, 16]. The observed modulation was likely due to a combination of a direct polarization of the cell membrane of CN cells, as the induced electric field (E-field) was significant at depths corresponding to the CN, and modulation of Purkinje cell (PC) activity, as PCs were strongly modulated, and they provide the large majority of synaptic input to CN cells [17, 18]. Which of these factors is dominant was not addressed systematically; however, at least with higher stimulus intensities, CN responses suggested direct polarization of the CN cell was a significant factor [15]. Here, we provide evidence that at least with transcranial placement of the electrodes, PC modulation is the primary driver of CN modulation.

More generally, whether and how PC and CN modulation induced by cerebellar surface and transcranial stimulation differs needs to be tested. Indeed, there are reasons to expect some differences. For example, interposition of the skull between the stimulus electrode and brain alters the pattern and amount of current flowing into the brain. Thus, the induced E-field within the brain is much weaker with transcranial as opposed to direct stimulation of the brain surface [19]. This raises the possibility that the entrainment patterns may vary significantly between the two stimulation methods. Thus, direct measurements are necessary to address if the cerebellar output can be modulated in a targeted way using transcranial AC stimulation.

Here, we show that ctACS causes a frequency-dependent modulation of PC and CN activity. Individual PCs showed a predominantly unimodal response to the AC stimulation, but at the population level, the response was bimodal. In contrast, most CN cells exhibited bimodal response patterns, particularly at the higher stimulus frequencies. Furthermore, the responses of the two cell populations support the hypothesis that ctACS modulates cerebellar output primarily as a result of PCs synaptically driving CN activity. The similarities and differences from brain surface stimulation are discussed.

## Materials and methods

Experiments were performed in accordance with the National Institute of Health *Guide for the Care* and Use of *Laboratory Animals*. Experimental protocols were approved by the Institutional Animal Care and Use Committee of New York University School of Medicine.

### Surgical procedures

Surgeries were performed on adult female Sprague-Dawley rats (n = 36). Rats had a mean weight of 259 ± 20.7 g and were 3–4 months old. Anesthesia was induced by an injection of ketamine (100 mg/kg) and xylazine (8 mg/kg) intraperitoneally. After the animal reached a surgical plane of anesthesia, a tracheotomy was performed to allow mechanical ventilation, if needed, and a femoral vein catheter was inserted to deliver supplemental anesthesia, given as a continuous intravenous injection of a ketamine-xylazine mixture (ketamine, 6μg/kg/min; xylazine, 1μg/kg/min). The depth of anesthesia was maintained such that pedal reflexes were absent throughout the experiment through perfusion at the conclusion of the recording session. Temperature was measured by a rectal probe and maintained at 37° C using a heating pad (TR-200, Fine Science Tools, Foster City, CA). Animals were placed in a stereotaxic frame. We targeted PC and CN cells (relative to Bregma, AP: −11mm, LAT: ±3.8mm). Burr holes (~ 1mm diameter) were made to allow access for a microelectrode to record spiking from PC and CN cells. Glass microelectrodes were systematically lowered from the cerebellar surface to isolate PCs (DV: 0 − 3 mm) and CN cells (DV: 3.5 − 4.5mm).

### Recording and stimulation procedures

ctACS was applied using Ag/AgCl electrodes, with one electrode on the skull surface overlying crus1 and one placed below the skin of the contralateral shoulder. Each ctACS electrode was constructed from a chlorided silver wire (diameter 0.015 “, #7830, A-M Systems, Sequim, WA, USA). The electrode’s end on the skull was shaped into a 2 mm diameter, 2–3 turn coil. The ctACS electrode wire was placed on the skull and soaked in electrolytic gel to ensure good contact. The indifferent electrode was placed in the shoulder region in the space between the muscles and the overlying skin and was ~ 5 mm in length. The indifferent electrode was made from silver wire (diameter 0.015 “, #7830, A-M Systems, Sequim, WA, USA).

Following placement of the stimulation electrodes, extracellular spike recordings were made with a NaCl (1 M)-filled glass microelectrode. Electrodes were inserted into the cerebellum using a joy-stick controlled micromanipulator (Burleigh Inchworm LSS-1000) to search for cells. Recordings were obtained from cells in the cortex and CN. Recordings were amplified with either a Dagan amplifier (Model EX1, 500–1000x gain) or an A-M systems AC amplifier (Model 1700 with 1000x gain) and filtered at 300 Hz and 10 kHz, and recorded using a multichannel recording system (MultiChannel Systems MCS GmbH, Reutlingen, Germany) with a sampling rate of 25 kHz/channel.

**Figure 1:**
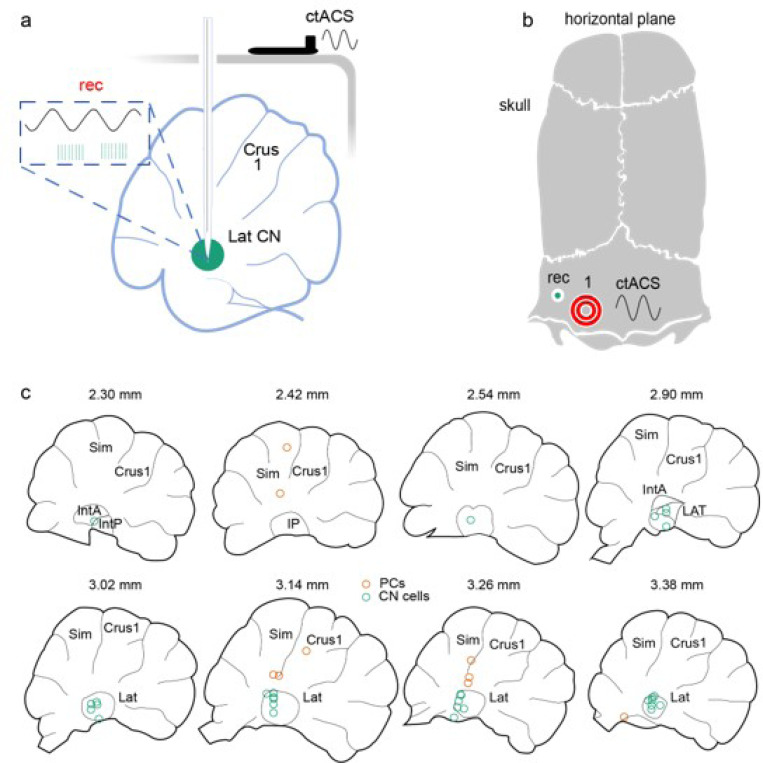
Diagram of experimental setup. **(a)** Sagittal schematic view of the cerebellum and overlying skull showing placement of ctACS electrodes directly on the skull above lateral crus1 and recording electrode inserted through burr hole to the CN. **(b)** Horizontal schematic of dorsal skull surface showing positions of the ctACS electrode on the skull above lateral crus 1 (red rings) and the burr hole for the recording electrode (green circle). **(c)** Schematics of parasagittal sections through the cerebellum showing the locations of histologically located PCs and CN cells (orange circles; n= 9, green circles; n= 30). Numbers above the sections indicate the mediolateral distance of the section from the midline.

The AC stimulation electrode was placed on the same side as the recording electrode in position 1, on the skull overlying crus1 and medial to the burr hole, relative to bregma, AP: −13.2 mm, LAT: ± 2.4 mm). (Fig. 1a-b); a mirror image configuration applies to recordings from the other hemisphere. In some experiments, a second stimulation site over the contralateral crus 1 was also tested. The stimulus peak-to-peak amplitude was 500 μA for all recordings. The AC frequency was systematically increased in steps from 0.5 Hz to 80 Hz. When a single unit was isolated, spiking activity was recorded for 3-min periods in which AC stimulation at a particular frequency was interleaved with periods of nonstimulation (10 s on/10 s off), allowing comparison of stimulus and spontaneous activity. AC stimuli were generated by an isolated current stimulator (Model SYS-A395D, WPI, Sarasota, FL) driven by a waveform generator (Model 185, Wavetek, San Diego, CA, USA). The following nominal frequencies were tested for all CN and 13/16 PC cells: 0.5, 5, 20, 50, and 80 Hz. Since the waveform generator had an analog frequency selector knob, stimulation frequency varied slightly from the nominal frequencies across recordings. Overall, the range of actual stimulation frequencies for each nominal frequency was: 0.5 Hz = (0.494-.514), 5 Hz = (4.99–5.14), 20 Hz = (19.2–20.0), 50 Hz = (49.9–51.2), and 80 Hz = (73.7–82.5). For simplicity, in the text and figure axis labels, the nominal frequencies are listed, but for statistical analyses in which stimulation frequency was an independent variable, the exact stimulation frequency was used.

### Data Analysis

Data files containing the extracellular recording traces, AC stimulus wave, and trigger times marking the start of the stimulation periods were converted to binary or text files using MC_Datatool software (MultiChannel Systems MCS GmbH) and then imported into Igor Pro 9 (Wavemetrics, Lake Oswego, OR, USA; RRID:SCR_000325). Data analysis was performed using built-in and custom-written routines with Igor Pro Microsoft, Excel, and R Project for Statistical Computing (RRID:SCR_001905).

To eliminate artifacts due to the AC stimulus, the raw recordings were high-pass filtered using a finite impulse response (FIR) filter with a Hanning window and 250–300 Hz cut-off (number of coefficients 701). Potential spikes were detected by either a single voltage threshold or two (upper and lower) thresholds with a time limit of 1 ms for crossing both thresholds. Next, principal components analysis (PCA) was performed on the detected spike waveforms, and a cluster plot of the first two principal components was used to isolate spikes from individual cells.

#### Calculation of circular mean of spike activity during AC stimulation.

The circular mean response vector was used as a non-parametric measure of the strength of modulation (i.e., degree of entrainment to the AC stimulation). To calculate the vector, every spike is represented as a unit vector in the direction equal to its phase with respect to the AC cycle and vectorially summed. This sum is then divided by the total number of spikes during the stimulation to get the mean vector. Notably, the mean vector length (r̄) can range from 0–1, where 1 indicates that the activity is perfectly phase-locked to the AC stimulation, and 0 indicates an entirely uniform distribution of phases (i.e., no phase-locking). In addition, the mean response vector’s phase (t̄) provides a measure of the average of the spike responses with respect to the AC stimulation.

#### von Mises-based models for separation of bimodal data into two spike populations.

Both uni- and bi-modal response distributions were observed in response to AC stimulation in CN cells. For unimodal distributions, the strength of modulation induced by ctACS can be directly characterized and quantified by the circular mean vector. However, bimodal distributions, particularly those in which the modes are ~ 180° out of phase, present a problem, as a near-zero magnitude circular mean vector can occur in such cases (because the spikes associated with one mode cancel the others’ contribution to the vector), implying the absence of modulation when in fact a strong modulation exists.

To deal with this situation, we devised a two-tiered approach in which responses are first classified as unimodal or bimodal, by fitting single and double von Mises functions to the phase with respect to the AC cycle of all spikes that occurred during the AC stimulation using the Mixtures of the von Mises-Fisher Finite Distributions (movMF) package in R (Foundation for Statistical Computing software 4.3.3). That is, the movMF algorithm was run with k = 1 and k = 2, where k sets the number of von Mises populations in the fit. The movMF package fit models to our spiking data (numerical matrix) using the expectation-maximization (EM) algorithm for maximum likelihood estimation [20]. We chose movMF because it provides probabilistic cluster assignments. Twenty EM runs were performed, allowing for more consistent results since the start of EM algorithm is random. We then compared the single and double von Mises fits using the Bayesian Information Criterion (BIC). A BIC difference of 6 or greater was treated as the boundary to favor a bimodal (k = 2) distribution [21]. Second, the final criteria for potential bimodal entrainment pattern based on the BIC difference, was the angle difference between the two von Mises populations: ≥ 50° was classified as bimodal (Fig. 3d, 6d) and < 50°was classified as unimodal.

The movMF package estimates the parameters of the von Mises population(s), the fraction of the total spike population represented by each von Mises distribution, and for every spike, the probability of it being in each von Mises distribution. (When k = 1, there is only one population, and so the probability is 1 for each spike; when k = 2, the sum of the two probabilities = 1).

For responses classified as unimodal, the circular mean vector was calculated directly from the data (i.e., every spike was equally weighted), as described earlier. For bimodal responses, two circular mean vectors were calculated, one for each of the identified von Mises populations. To do so, each individual spike vector was weighted by the probability that it belonged to the particular population in the vector sum. Although the circular mean vector is a non-parametric statistic, the validity of our division of bimodal responses into two spike populations rests on the goodness of the single and double von Mises fits to the data. Thus, the Kolmogorov-Smirnov (KS) test was used to evaluate whether the fitted data reflected the observed entrainment data. Specifically, a histogram was generated of the phases from all spikes totaling 30 bins and compared with the predicted values from the von Mises fit using the KS test.

#### Calculation of population vectors.

To provide an overall measure of a particular cell type’s response to AC stimulation, a population vector was calculated as the vector sum of the individual cell circular mean vectors weighted by their r̄ ‘s and alphas divided by the number of cells in the sum.

### Statistics

Unless stated otherwise, t-tests, paired or unpaired, or ANOVA, as appropriate, were used to test for statistical significance based on the built-in functions in R and libraries built for circular statistics [22]. Mixed models were chosen to handle unbalanced data and to treat frequency as a continuous variable. We modeled the cell as a random effect across frequency. A general linear mixed-effects (GLMM) and a linear mixed-effects (LME) model were used with the lme4 (RRID:SCR_015654) [23], to calculate Wald chi-square or F test values using lmerTest [24].

To assess whether a cell’s activity was significantly modulated, we used the Rayleigh test for uniformity for circular data and the Rao spacing test for multimodal distributions. All responses that were found to be non-uniform by at least one of these tests were considered to show evidence of modulation by the AC stimulus, and were then classified as unimodal or bimodal, and analyzed as described earlier. Circular mean values are presented with their circular standard deviation (sqrt(−2lnr)) in the text [25]. Other than for circular data, means are given with their standard deviation (SD).

#### Histological and stereotaxic procedures for identifying cell locations.

After a recording track was completed, alcian blue dye was injected in the middle of the track (depth ~ 2 mm) and at the end of the track (depth ~ 4 mm). At the conclusion of the experiment, the animal was perfused with normal saline followed by 10% formalin. The brain was removed, soaked in 10% formalin for at least 1–2 days, and then transferred to a 30% sucrose/10% formalin solution until it sank. Parasagittal 60-μm sections of the cerebellum were prepared on a freezing microtome and mounted on chrome–alum gelatin-coated slides for counterstaining with cresyl violet. To identify cell locations, tissue shrinkage was calculated by measuring the distance from the brain surface at the electrode entry point to the dye spots and taking the ratio of this measurement to the micrometer value of the dye spots. Micrometer values for the depths of recorded cells were then reduced by this factor and then plotted along the track. Typically, tissue shrinkage was ~ 10%.

In experiments where multiple tracks were made, dye spots were only made on some tracks to avoid ambiguity in defining each track. In these cases, when cells were recorded on unmarked tracks, and their location was defined using shrinkage-corrected micrometer readings and comparison to the nearest dye-labeled track. In addition, cells recorded at depths less than < 3.0 mm were categorized as cortical neurons, as the shortest distance from the surface of the crus 1 or paramedian lobules to the most superficial portion of the cerebellar nuclei is ~ 3 mm.

## Results

**Figure 2:**
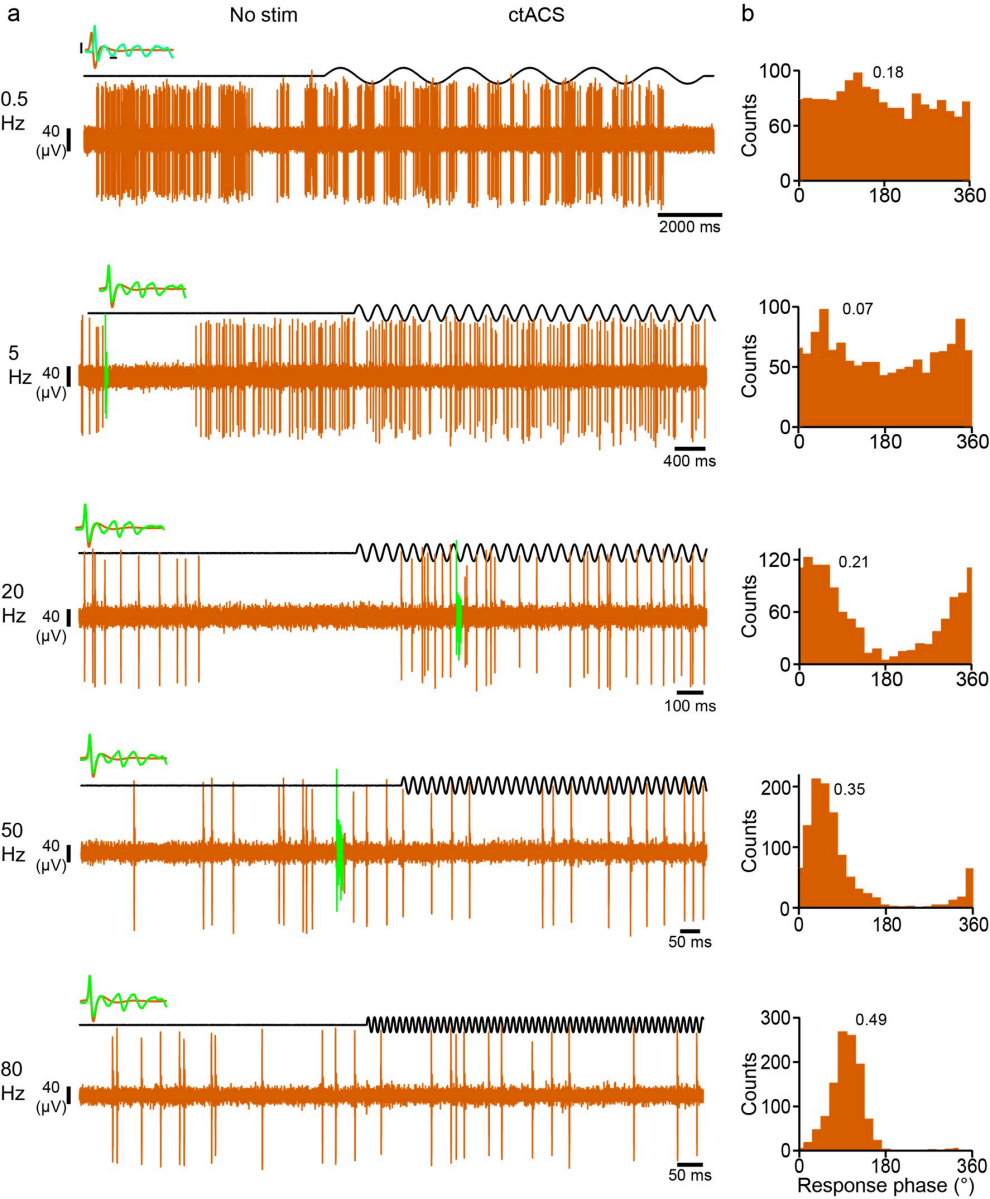
Unimodal entrainment of PC activity. **(a)** Example extracellular recordings from a PC (orange trace) in response to interleaved periods of no stimulation and AC stimulation (black trace) for frequencies of 0.5, 5, 20, 50, and 80 Hz (top to bottom). Inset, example waveforms corresponding to each recording (SS, orange, CS green; black bars = 10 μV, 0.5 ms). **(b)** Histograms of (PC SS) activity with respect to the AC cycle for frequencies 0.5 (top) through 80 Hz (bottom). Histograms of spike activity triggered off the start of each sinusoidal cycle, and bins were set to 1/20 of the cycle period. The r̄ for each frequency is shown as text.

Previous studies showed that a clear and robust entrainment of PC and CN cell activity occurs when AC stimulation is applied directly to the cerebellar cortex [15, 16, 26]. To assess the entrainment pattern of PC and CN cells when AC stimulation is instead applied to the external surface of the skull. This configuration that more closely matches the situation in transcranial stimulation (Fig. 1a-b), we applied AC stimulation at frequencies, ranging from 0.5 to 80 Hz, to the skull surface above crus 1, and recorded single unit spiking from a total of 64 cells: non-identified cortical cells (n = 15), PCs (n = 16) and CN cells (n = 33; lateral CN: n = 27; AIP: n = 3, PIP: n = 3) (Fig. 1c). The locations of the recorded cells were histologically identified (Fig. 1c). In addition, PC recordings were identified as such by the presence of complex spikes (CS) (Fig. 2a, green traces) and the CS-induced pause of simple spikes (SS) (not shown). Recordings at each AC frequency consisted of approximately ten 10-s long periods of AC stimulation interleaved with 10-s nonstimulation periods during which spontaneous activity was recorded, for a total recording period of ~ 3-min at each stimulus frequency.

### Frequency-dependent entrainment of PC SS spiking.

PC SS’s average spontaneous and stimulated firing rates (FR) collapsed across stimulus frequencies were not different (spontaneous, 21.3 ± 5.90; stimulated, 19.8 ± 5.12 Hz; n = 12 rats; *p* = 0.33), respectively. In addition, no difference in firing rate was found at any stimulus frequency (LMER model: (FR ~ NS_S * stimulus frequency [*F*_1,156_ = 0.410, *p* = 0.52]). Despite little or no change in average FR, PCs showed strong modulation of their SS activity. Histograms of PC SS activity triggered off of the start of the AC cycle showed that AC stimulation modulated SS firing rates in the majority of PCs at the lower (0.5 Hz and 5 Hz) frequencies and in all PCs at the higher (20 Hz − 80 Hz) frequencies. Moreover, the strength of the modulation varied with stimulus frequency, being relatively weak at lower frequencies, with a robust entrainment emerging as frequencies increased from 20 to 80 Hz, as shown by the histograms of SS activity for an example PC during AC stimulation at each frequency (Fig. 2b).

**Figure 3:**
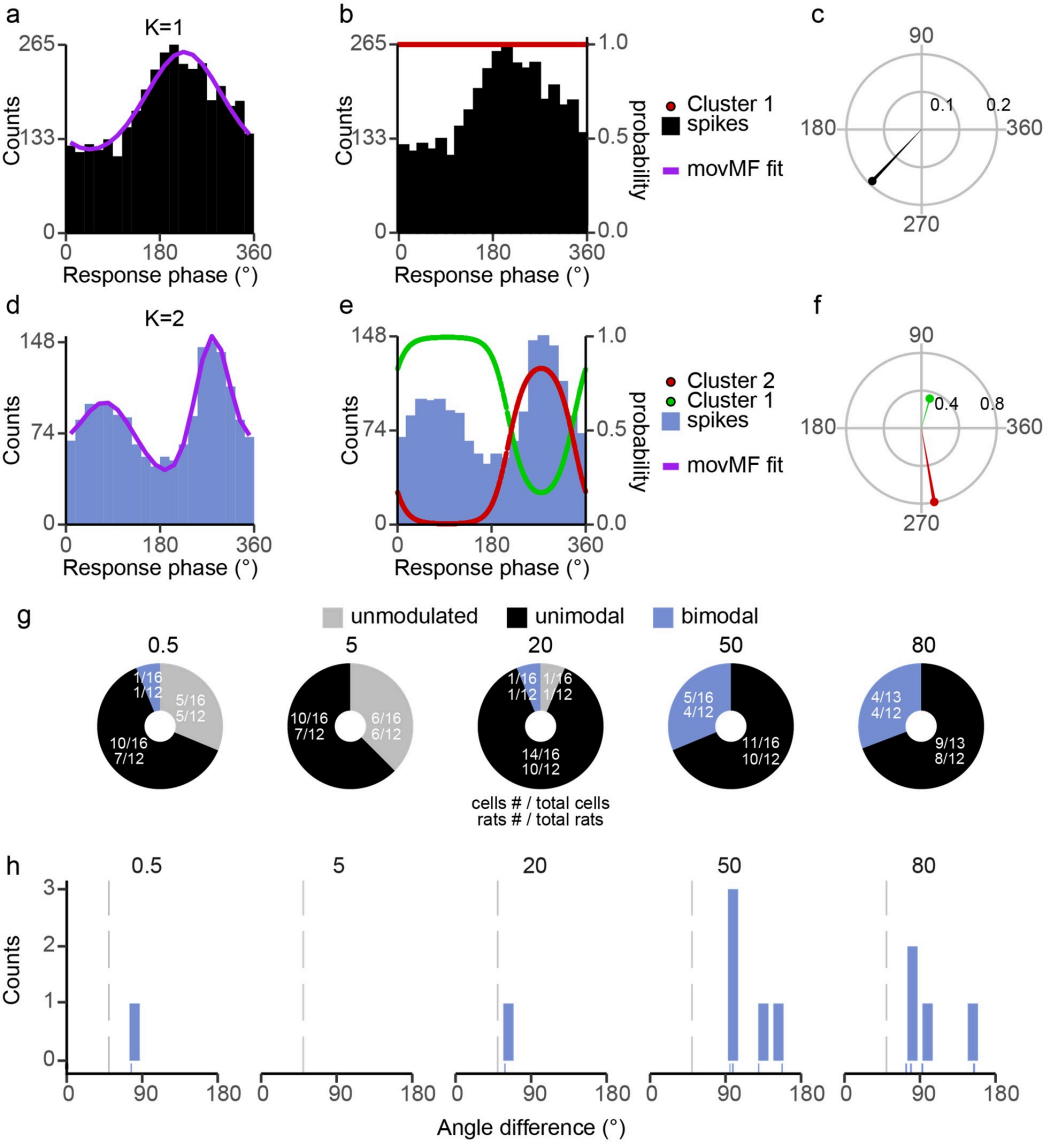
Quantification of PC SS modulation. **(a)** Example fit of unimodal distribution. Histogram of phase distribution of SS activity during AC stimulation at 50 Hz. For this cell, a single von Mises function (K=1, purple) was found to be the best fit. Histogram bins were set to 1/20 of the cycle period. **(b)** The histogram in 3a is replotted along with the probability of each spike being in the single von Mises population (K=1) plotted as a function of phase (red circles, right axis). Note population is fit by a single distribution, so probability always equal one. **(c)** A polar plot of the circular mean vector for the spike data from 3ab. **(d)** Example fit of a bimodal distribution in which a mixture of two von Mises functions was found to be the best fit (K=2), same conventions as 3a. **(e)** Same as 3d, except the probabilities for the two clusters are shown (k=2). **(f)** For bimodal cells, the circular mean vectors for each von Mises population are plotted. **(g)** Donut plots show the number of PC cells/rats for each entrainment class across frequency. Unmodulated (gray); significantly modulated: unimodal (black) or bimodal (blue). **(h)** Histogram of the minimum angle difference between circular mean vectors. For most bimodal cells the angular difference is near 90°.

Because of the complexity of PCs showing uni- and bi-modal entrainment patterns, a multistep approach was used to characterize the PC entrainment patterns and categorize their distribution (see Methods). Figure 3 illustrates this process. The histograms in Fig. 3a-b show a typical unimodal response pattern, whereas a bimodal response pattern is shown in Fig. 3d-e. The distribution for each PC was not significantly different from its fit (Fig. 3a and 3d). The probability of each spike belonging to the fitted von Mises distributions are plotted with the same two histograms in panels B (k = 1, red data points, right axis) and E (k = 2, red and green data points, right axis). The polar plots in Fig. 3c and 3f show the circular mean vectors for the two PCs. In the example shown in Fig. 3f, the direction of the two mean vectors was approximately 154.4° apart. Unimodal PCs are the dominant mode of entrainment at each frequency (Fig. 3g). Furthermore, the number of significantly modulated cells increases with frequency (Fig. 3g). For bimodal PCs, the angle difference ranged between 90° and 180° at higher frequencies (Fig. 3h).

**Figure 4:**
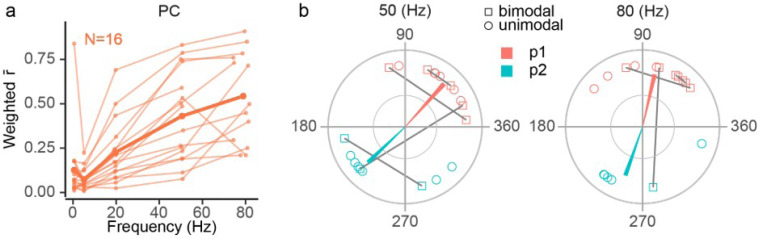
PC modulation is frequency dependent. **(a)** Each PC’s weighted circular mean vector length (r̄) is plotted as a function of AC frequency. The bold trace reflects the population mean. **(b)** For 50 and 80 Hz, plots showing the phase of each PC’s circular mean vector (open circles are unimodal, open squares are bimodal) and the population vector for each cluster (p1 and p2) were calculated as a weighted average of the individual vectors (weighted the magnitude and population fraction of the von Mises population). The dark gray lines connect the two phases of bimodal cells.

To quantify the entrainment strength of PC modulation across frequencies an overall r̄ was calculated for each PC at each stimulus frequency. For unimodal cells, this was simply r̄ calculated as described in the Methods. For bipolar PCs, the overall r̄ was calculated as the weighted (by alpha) average of the r̄ ‘s (again calculated as described in the Methods) of the two von Mises populations that were fit to the PC’s response. An increase in modulation strength with increasing AC frequency between 0.5 and 80 Hz stimulation was observed in most PCs (Fig. 4a, GLMER model: r̄ ~ stimulus frequency [*x*^2^_,2_ = 179.7, *p* < .001]). Most cells showed larger r̄ ‘s at 50 Hz and 80 Hz than at 0.5–20 Hz; however, in some cases, the individual cell r̄ curves can be seen to plateau or even decline slightly between 50 Hz and 80 Hz, suggesting that the optimal frequency for modulating PCs is around 80 Hz or slightly higher. Indeed, for stimulation applied directly to the cerebellar cortex, modulation strength was shown to peak at frequencies around 100 Hz [27].

We compared the t̄‘s for the higher stimulus frequencies to investigate the timing of the PC population response when PCs were strongly modulated (i.e., 50 Hz and 80 Hz, Fig. 4b). As a population (both unimodal and bimodal cells are plotted), t̄ values of the SS activity formed two clusters, one during the positive phase of the AC cycle, and one during the negative. Note that bimodal cells are plotted with two points to show the phases of both modes. We calculated population vectors for each t̄ cluster by summation of the individual cell circular mean vectors after weighting each vector by its alpha. Of note, the phase of the population vector for each cluster showed little change between 50 to 80 Hz for population 1 (Fig. 4b: Wilcoxon two-Sample T-test [*T* = 45, *p* = 0.182], 48.0° to 77°) and population 2 (Fig. 4b: Wilcoxon two-Sample T-test [*T* = 130, *p* = 0.087], 224° to 251°).

In sum, the data shows that AC stimulation applied directly to the skull causes in most PCs a unimodal, frequency-dependent entrainment; however, at the population level SS modulation is bimodal primarily because of the differing phases of the individual PC activity.

### CN cells show bimodal frequency-dependent Modulation during AC stimulation.

**Figure 5:**
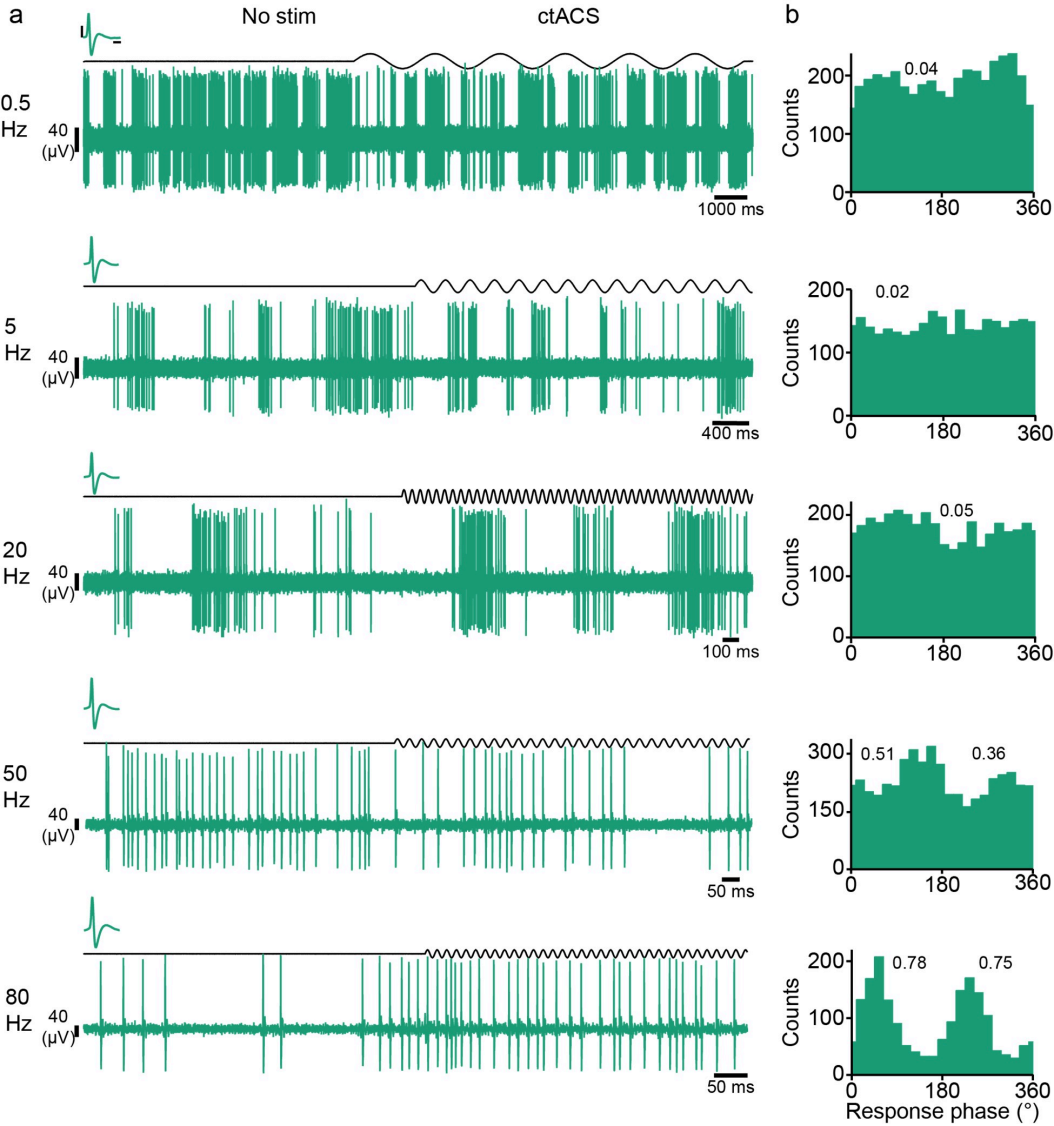
CN modulation is frequency dependent. **(a)** Example extracellular recordings from a Lat CN cell (green traces) in response to interleaved periods of no stimulation and AC stimulus train (black traces) for frequencies 0.5 (top) through 80 Hz (bottom). Inset, waveform of CN spikes (black bars = 10 μV, 0.1 ms). **(b)** Histograms of Lat CN cell activity with respect to the AC cycle for frequencies 0.5 (top) through 80 Hz (bottom). The r̄ for each frequency is shown as text.

The ability of ctACS to modulate SS activity suggests that CN cells, which are downstream to PCs, are also likely to be affected. To investigate this possibility, we recorded single-unit CN cells in the lateral (Lat) CN and applied the same AC stimulation protocol as was used for the PC recordings (Fig. 5a). Lat CN cells were identified based on the following criteria: (1) The recording depth (3.3–4.5 mm) from the cortex surface (n = 1) and/or (2) cell location within the CN based on tracks reconstructed from histological parasagittal sections (n = 32). These cells showed spontaneous bursting or tonic firing patterns typical of previous descriptions of CN activity [28]. The average CN spontaneous firing and the stimulated rate were 44.6 ± 16.7 Hz and 43.0 ± 15.3, respectively (n = 16 rats, LMER model: (FR ~ NS_S * stimulus frequency [*F*_1,156_ = 0.410, *p* = 0.52]).

Similar to PCs, the modulation of CN neuron spiking increased with frequency over the tested range, which is most easily seen in the histograms triggered off of the start of the AC cycle (Fig. 5b). Again, unimodal and bimodal response patterns were found.

**Figure 6:**
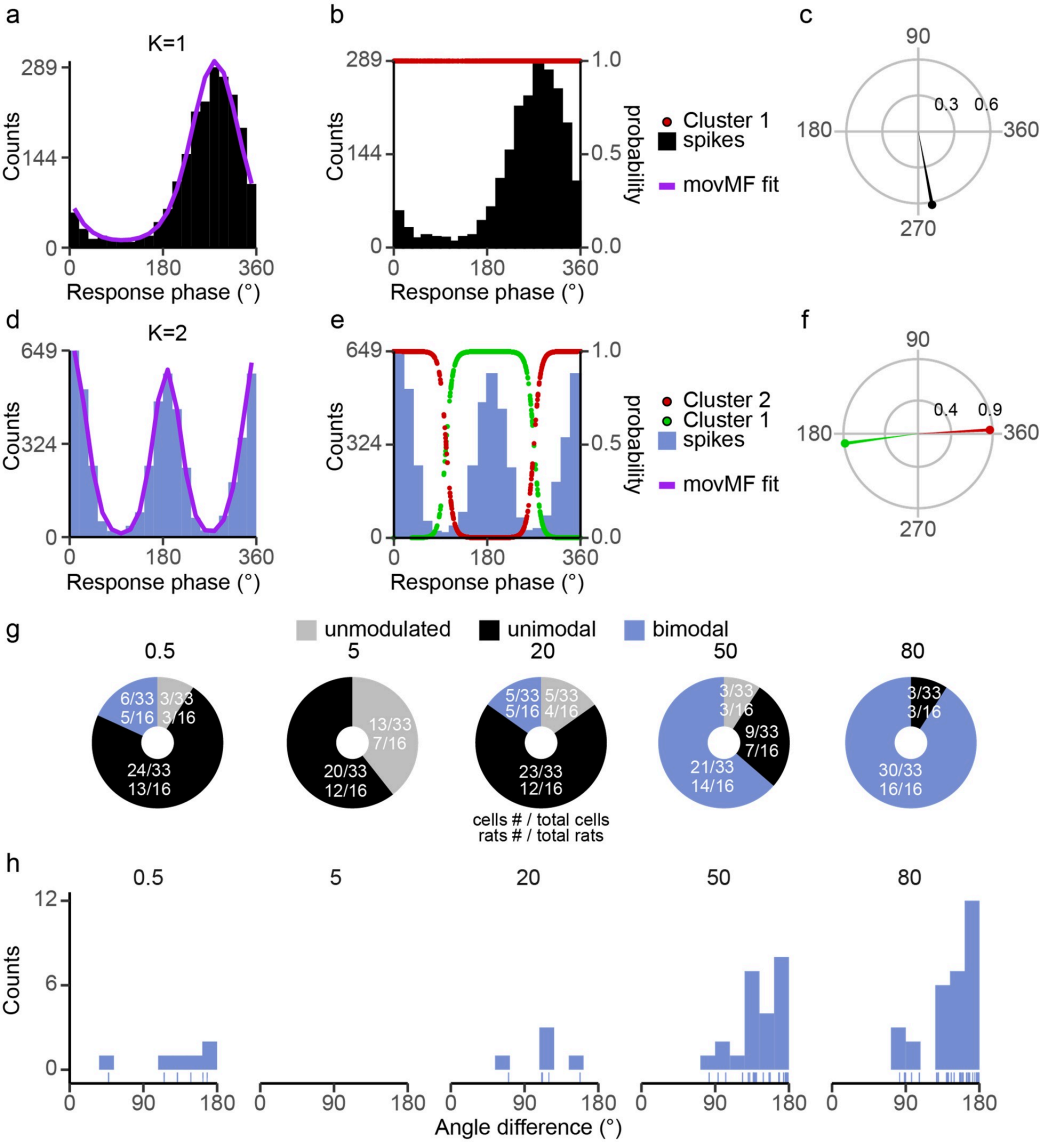
Quantification of modulation strength for CN cells. **(a)** Histogram and single von Mises fit (purple) to aCN cell with a unimodal distribution with 50 Hz ctACS. **(b)** Replotting of histogram in 6a along with probability of each spike belong to the particular von Mises distribution (red circles, right axis). **(c)** Circular mean vector for the unimodal CN cell in 6ab. **(d)** Histogram and mixture of two von Mises fit to aCN cell with a bimodal distribution during 50 Hz ctACS. **(e)** Replotting of histogram in 6d along with probability distributions for the two von Mises populations (green and red circles, right axis). **(f)** Circular mean vectors for the two von Mises spike populations of the CN cell shown in 6de. **(g)** Donut plots show the number of CN cells/rats for each entrainment class across frequency. Unmodulated (gray); significantly modulated: unimodal (black) or bimodal (blue). **(h)** Histogram of the phase differences between the two circular mean vectors of all bimodal CN cells, showing most bimodal cells tend to be close to symmetric (~180°).

Our approach to classifying and analyzing the response distributions was the same as was done with PCs and is shown in Fig. 6, which compares two CN cells that were classified as having unimodal (Fig. 6a-c) and bimodal (Fig. 6d-f) responses to ctACS. The unimodal histogram has a peak response at 280.6° (Fig. 6a), whereas the bimodal has peaks at 2.9° (spanning the start and end of the AC cycle) and one at 187.6°. The single (Fig. 6a) and double von Mises (Fig. 6d) fit closely following their respective histograms, suggesting that CN responses follow a single or double von Mises distribution, as did PCs. Figure 6b and 6e replicate the histograms in 6a and d along with the probabilities of being part of each population for all spikes (red and green circles, right-hand axis). Figure 6c shows the single circular mean vector for the unimodal CN cell. Figure 6F shows the two circular mean vectors for the bimodal CN cell, which were calculated using the probabilities shown in Fig. 6e to weigh each spike’s contribution to the mean circular vector.

ctACS was found to significantly modulate CN activity (Fig. 6g). Moreover, CN cells could transition from a unimodal response to a bimodal one at higher frequencies (Fig. 6g). Unlike PCs, the bimodal pattern was the dominant mode of entrainment of CN cells for 50 and 80 Hz, while the unimodal pattern was the main mode of entrainment at lower frequencies, 0.5–20 Hz (Fig. 6g). In addition, the phase difference between the two peaks in the bimodal responses varied between cells. Furthermore, the phase of the peaks shifts with frequency, which will be described in detail below.

In the example shown in Fig. 6f, the direction of the two mean vectors was approximately 180° apart; however, this was not always the case. The distribution of the phase differences between the two vectors calculated for each bimodal response is shown across stimulation frequency in Fig. 6h. Overall, the distribution at all frequencies was similar, with most bimodal CN cells showing a phase difference between ~ 90° to ~ 180°, with a difference close to 180° being the most common pattern. However, the 80 Hz distribution is somewhat right shifted compared to the 50 Hz one.

**Figure 7:**
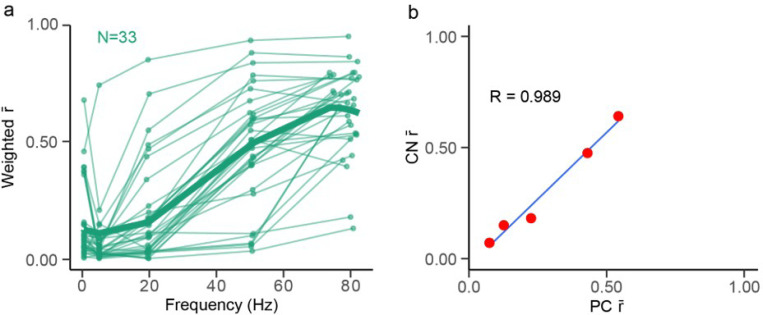
CN modulation increases with frequency. **(a)** Individual CN cell r̄ plotted across frequency for each CN cell. For cells with bimodal responses, the average r̄ is the weighted average of the r̄ ‘s of the two von Mises populations (weighted by their alphas). The bold green trace is the predicted value from our statistical model. **(b)** A plot showing the linear relationship in modulation strength (r̄) between PC and CN populations, the blue line indicates the best-fit line.

To determine how modulation strength varies with frequency, we plotted each CN cell’s overall r̄ (average of the two von Mises r̄ ‘s weighted by their alphas) as a function of frequency (Fig. 7a. Most cells showed weak or non-significant modulation at the lower frequencies, and stronger modulation at higher frequencies, as measured by overall r̄. For most CN cells (28/33), r̄ generally increased with frequency between 5 Hz and 80 Hz, although in a minority of cases (5/33), r̄ decreased between 50 Hz and 80 Hz (Fig. 7a: LME model: r̄ ~ stimulus frequency [*F*_3, 129.3_ = 128.35, *p* < .001]). This suggests that around 80 Hz is close to the frequency for maximal modulation of CN activity, paralleling what was seen with PCs. Results from another study in which direct brain surface stimulation at higher frequencies was tested showed reduced entrainment CN activity above 100 Hz [27], providing further evidence that frequencies around 80–100 Hz are optimal for driving CN activity. We assessed the relationship in modulation strength (r̄) between CN and PCs as a function of frequency. We found the r̄ of the PCs and CN cells with stimulus frequency was highly correlated (Fig. 7b, R = 0.989, t_,3_ = 11.9, *p* < .001).

### Population-level entrainment for CN spiking.

**Figure 8:**
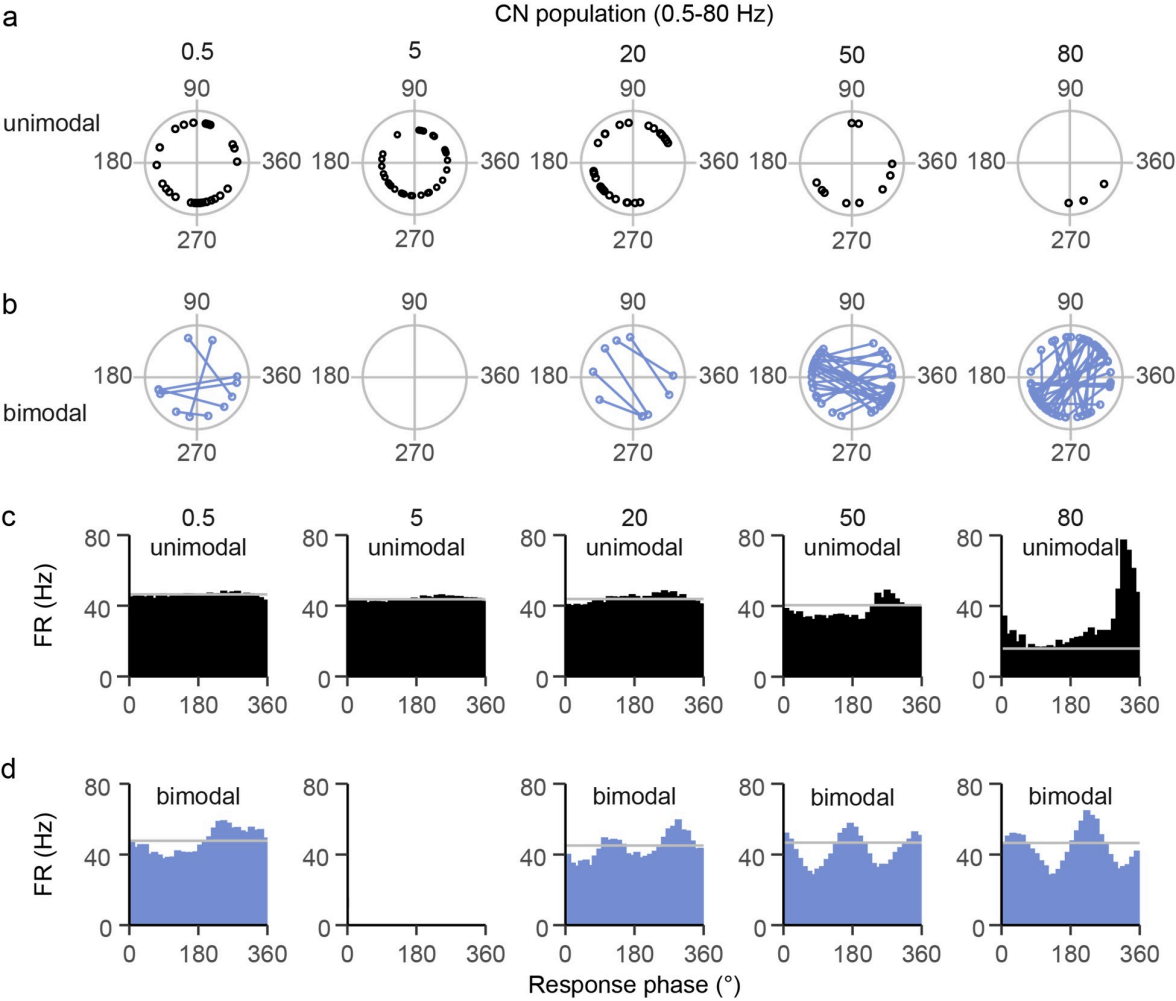
CN population changes from a unimodal to a bimodal response with increasing AC frequency. **(a)** distribution of CN circular mean vector phases (open circles) is plotted for cells that showed an unimodal pattern at 0.5 to 80 Hz. **(b)** Same as ‘a’ for bimodal cells. The two vector phases of each cell are connected by a line. Note the transition from mostly unimodal cells to mostly bimodal cells as frequency increases. **(c)** Histograms showing the population modulation for unimodal cells in response to different AC frequencies. The gray line indicates the average spontaneous firing rate at each frequency. **(d)** Histograms showing the population modulation for bimodal cells in response to varying AC frequencies. The gray lines indicate the average spontaneous firing rate at each frequency. Note that no CN cells showed a bimodal response to 5 Hz stimulation.

We investigated the main entrainment pattern in our entire CN dataset to understand what occurs at the Lat CN population level during robust AC entrainment, and thus reflects the cerebellum’s output. Specifically, we determined the phase distribution of the mean circular vector during 0.5 to 80 Hz AC stimulation across the population. The phases of the vectors for both CN response classes are plotted in Fig. 8a and 8b. At the lowest frequencies (0.5 Hz and 5 Hz), the individual cell t̄ ‘s of the unimodal cells have a widespread distribution of phases (Fig. 8a: Rayleigh test of uniformity: 0.5 Hz, *p* = 0.153, 5 Hz, *p* = 0.230). Moreover, the r̄ ‘s at these frequencies is relatively low. As a result, histograms of activity from the entire unimodal CN population show little to no modulation at these frequencies about the spontaneous firing rate indicated by a gray line (Fig. 8c). As the AC frequency increases the unimodal vector distribution becomes less uniform and the individual cell r̄ ‘s increase, resulting in a clear modulation with 50 Hz and 80 Hz stimulation. However, relatively few CN cells showed a unimodal response at these higher frequencies and instead showed a bimodal response.

In contrast to the unimodal responses, bimodal responses produced a significant modulation of CN activity at the population level at all frequencies, except at 5Hz when no CN cells showed a bimodal response, which grew stronger with increasing AC frequency (Fig. 8d). The population modulation was due to the non-uniform distribution of the individual cell vectors, as seen in the polar plots of Fig. 8b (lines connect the phases of the two vectors for each cell).

In sum, CN neurons as a whole show unimodal or bimodal entrainment pattern across the range of tested AC frequencies, with the modulation becoming stronger and the bimodal pattern becoming dominant as the stimulus frequency increases.

### CN entrainment is synaptically driven.

The results presented thus far show that CN activity can be significantly modulated by AC stimulation applied to the skull surface, which raises the question of the underlying mechanism: is CN modulation primarily due to direct polarization of a CN neuron by the external E-field or is it driven by modulation of the PC activity? Given the depth of the CN from the cerebellar surface, one would assume that the E-field in the CN would be weak relative to that in the cerebellar cortex, suggesting that CN entrainment would primarily be due to synaptic driving by PCs, the primary input to the CN. However, when AC stimulation is applied to the cerebellar surface directly, the resulting E-field can be significant at depths corresponding to the CN, raising the possibility of direct effects on CN neurons [15]. Thus, we sought evidence that with ctACS, synaptic driving of the CN by PCs is the primary mechanism for CN modulation.

**Figure 9:**
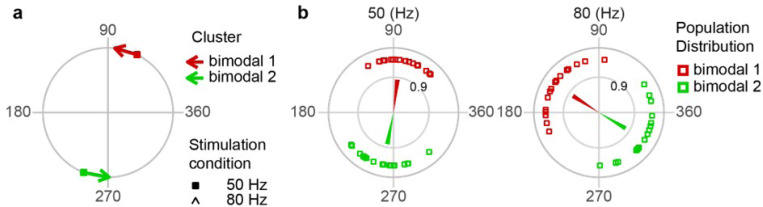
CN population minimum rotates with AC frequency. **(a)** The minimum counterclockwise rotation rule (red is cluster 1, green is cluster 2) that was used to identify and track each minimum across frequencies in the bimodal cells whose von Mises fits showed two minima (filled square, phase at 50 Hz; arrowhead, phase at 80 Hz). **(b)** Plots of the phases of the individual cell minima (open red and green squares) at 50 and 80 Hz. Also plotted are the population minima vectors. Note the rotation of the phase distributions and vectors between 50 and 80 Hz.

Comparison of the polar plots and the histograms at 20, 50, and 80 Hz in Fig. 8a and 8b provides evidence consistent with synaptic driving of the CN: the phase of the individual circular mean vectors and the population vectors computed from them rotate in the counterclockwise direction between 50 Hz and 80 Hz. This rotation is consistent with a constant delay in the effect of the E-field on the CN cells, which would cause a greater phase shift at the higher frequency. The most likely source of such a delay is the conduction time of action potentials down the axons of PCs. To investigate this quantitatively, we first note that PCs are inhibitory and so would produce a decrease in CN firing. Thus, for each bimodal CN cell we found the local minima in its fitted double von Mises function. The large majority of bimodal cells had two local minima at both 50 Hz and 80 Hz, and the phases of these minima are plotted in Fig. 9. Based on their clustering at 80 Hz, the frequency with the strongest modulation, the CN cells were divided into two clusters (green and red squares). To match corresponding troughs across frequencies, a minimum counterclockwise rule (going from 50 Hz to 80 Hz) was used (Fig. 9a), and resulted in a clear clustering of the CN cells at 50 Hz (Fig. 9b). (The counterclockwise rule is justified by the fact that a constant time delay leads to a larger phase shift as stimulus frequency increases.) To calculate the mean population trough, the individual cell troughs were weighted by the modulation strength of the cell (the weighted average of the r̄ ‘s of the two von Mises populations, where each r̄ was weighted by its alpha, the fraction of the total spike population) and vectorially summed. These mean trough vectors rotate 83° to 147° (bimodal 1) and 259° to 329° (bimodal 2) between 50 Hz and 80 Hz bimodal 1 (Fig. 9b: Wilcoxon two-Sample T-test [*T* = 447, *p* < 0.001]) and bimodal 2 (Fig. 9b: Wilcoxon two-Sample T-test [*T* = 399, *p* = < 0.001]), which is consistent with the E-field acting at a constant time delay on CN cells.

**Figure 10:**
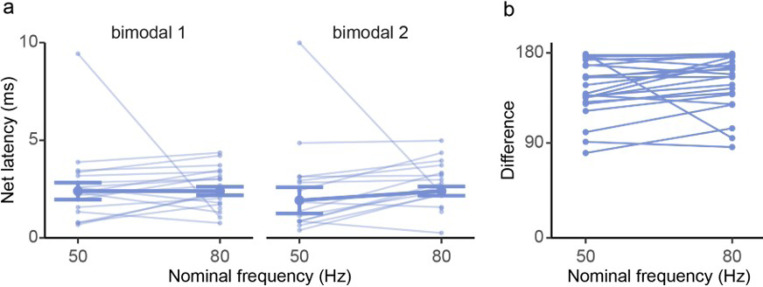
CN latency is consistent with synaptic modulation by PCs. **(a)** The calculated latency of the minima in the CN response distribution relative to the phase of the PC peak response for each bimodal CN cell (light blue traces). The means population latency is shown as a dark blue trace. **(b)** The line plot shows the calculated minimum angle difference between clusters for each bimodal CN cell at 50 and 80 Hz, n=25.

If CN cells are being driven from modulated PCs, the latency between the peak responses of the PCs (their t̄ ‘s) and the troughs of the CN responses should correspond to the known conduction time from PCs to CN cells in rats, 2.31 ± 0.92 ms [16]. We don’t know the peak response phases of the specific PCs projecting to each recorded CN cell, so the peak PC responses were estimated using the phases of the two PC population vectors at 50 Hz and 80 Hz (Fig. 4b). The phase difference between each trough in the CN response and the closest preceding PC population peak response was converted to the time domain by multiplying the period of the AC cycle and dividing by 360° to get an estimate of the latency. The calculated mean latency for all bimodal CN cells is 2.55 ± 1.46 ms (Fig. 10a), which is essentially identical to the measured PC-CN conduction time cited earlier (One-Sample T-test: *t*_31_= 1.16, *p* = 0.25).

These latencies can be used to test whether different PC populations are driving the CN cell at various frequencies, which could contribute to the changing strength of the modulation across frequencies. The latencies were stable across 50 and 80 Hz (Fig. 10a: LME model: Ct ~ stimulus frequency + Cluster [*F*_1, 59_ = 0.002, *p* = 0.96]). Furthermore, we calculated the phase difference between the two trough vectors of each bimodal CN cell at 80 Hz and 50 Hz to assess whether the vectors rotated similarly with frequency. The vast majority of CN cells had similar phase differences at 50 Hz and 80 Hz (Fig. 10B: paired t-test: *t* = 1.19, df = 91, *p* = 0.236). Thus, if any additional PCs that were recruited at 80 Hz, they had, on average, similar conduction times to those at 50 Hz. Alternatively, the increase in CN modulation between 50 Hz and 80 Hz may simply reflect an increase in the modulation of the PCs already being modulated at 50 Hz.

**Figure 11:**
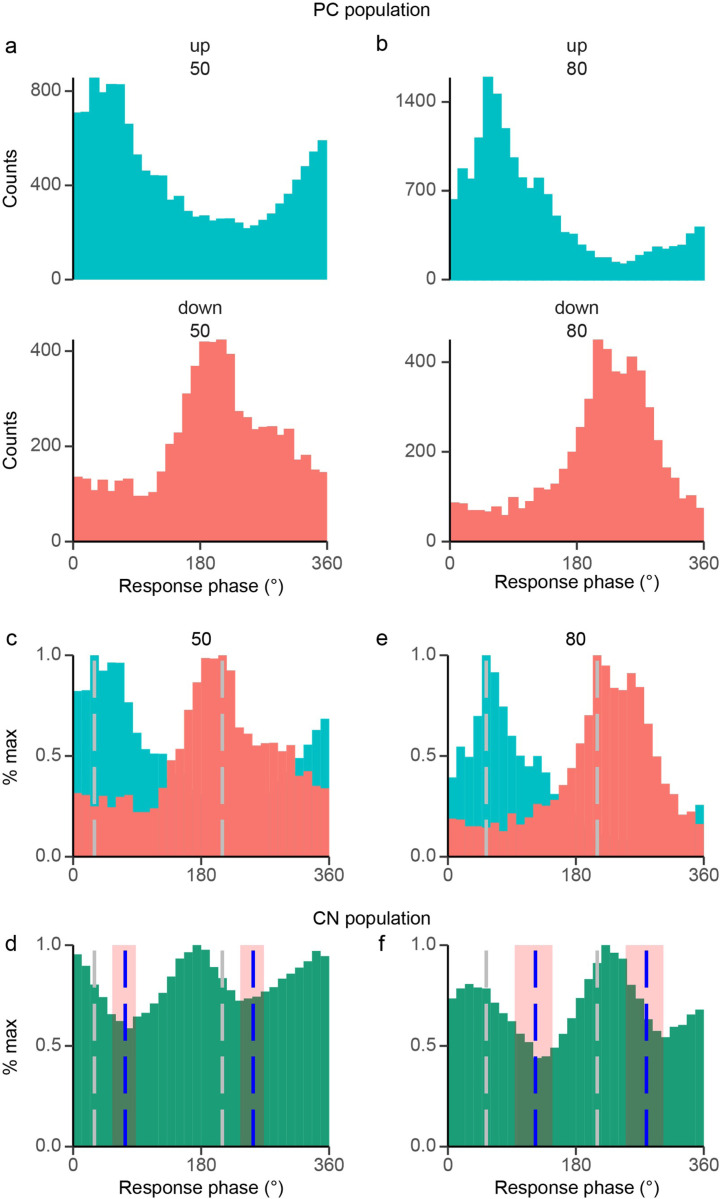
PC population response is consistent with driving CN bimodal responses. **(a)** At 50 Hz, histograms of the PCs with circular mean vectors with phases 0° - 180° (up PCs, top) and phases 180° - 360° (down PCs, bottom). **(b)** Same as 11a but for 80 Hz stimulation. **(c)**Overlaying the two up and down PC populations histograms makes a bimodal distribution at 50 Hz. The gray dashed lines indicate the peaks of each unimodal PC distribution. **(d)** Population histograms of bimodal CN cell responses to 50 Hz. **(e)** Same as 11c but for 80 Hz. **(f)** Same as 11d, but for 80 Hz. For 11d and 11f, the blue dashed lines indicate the predicted time of maximal inhibition of CN activity based on the mean latency of 2.55 ms from the peak PC population response. The light red rectangles indicate the expected range of phase shifts using the reported PC - CN conduction times of 1.39 – 3.23 ms.

We evaluated whether the PC population can explain CN’s bimodal nature to probe the PC-CN synaptic hypothesis further. We pooled PCs by whether their circular mean vector occurred in the positive phase (up PCs, Fig. 11a, top) or the negative one (down PCs, Fig. 11a, bottom). The two PC groups were combined for each frequency, showing that at a population level PC output is bimodal, unlike the case for most individual PCs, which have unimodal responses. The two population histograms are merged in Fig.s 11c and 11e, where gray dashed lines show the peaks of the PC populations. The bottom row (Fig. 11d and 11f) shows the population CN response to AC stimulation at 50 and 80 Hz. The gray dashed lines again indicate PC peak activity and the calculated conduction times from this activity (blue dashed lines) occur at the troughs in the activity. The known conduction time from PCs to CN cells ranges from 1.39 to 3.23 ms, which is overlayed on the bimodal CN populations (pink square) (Fig. 11d and f). Notably, the calculated and reported latencies generally cover the troughs in CN spiking for 50 and 80 Hz, as PC synaptic input is inhibitory.

Collectively, the rotation of the local minima with frequency is consistent with and is expected if CN cells are being driven synaptically, falling within the known conduction time from PCs to CN cells (~ 1.39–3.23 ms). The fact that the latencies do not increase at 50 and 80 Hz suggests that the same populations drive the CN cells, so it is likely that a more robust modulation of the same PC populations will lead to a stronger CN modulation. Thus, as a population, PC responses to AC stimulation are appropriately timed to underlie the modulation of CN activity.

### ctACS location modulates CN entrainment strength.

**Figure 12:**
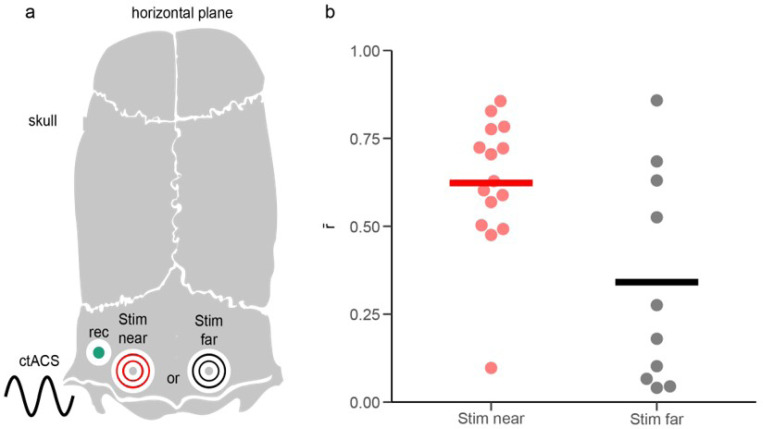
Entrainment strength depends on ctACS location. **(a)** Diagram of dorsal skull surface showing positions of AC electrodes relative to the recording site. Position one is adjacent to the recording site (Stim near), and position two is over the contralateral cerebellum (Stim far). **(b)** The distribution of r̄ ‘s for each position, with horizontal bars representing the mean.

The results presented thus far all showed responses to AC stimulation applied to the skull directly over the Lat CN and the region of cortex that projects to it. However, stimulus electrode location may be an important parameter for localizing and driving CN entrainment with ctACS, particularly as the skull likely causes a wider, albeit weaker, E-field distribution in the cerebellum. To investigate this issue, two stimulation electrodes were placed at different skull positions. The first electrode was positioned ipsilaterally, just medial to the recording track, while the second electrode was positioned on the contralateral side of the skull (Fig. 12a; a mirror image arrangement was used for recordings from the right cerebellum). Responses to both stimulation electrodes were compared at a frequency of 80 Hz stimulation in 8 CN cells, as this frequency produced the most robust modulation. Most CN cells showed significant modulation to both stimulus sites (ipsilateral, 8/8 cells; contralateral, 8/8 cells). However, ipsilateral stimulation produced a significantly stronger modulation (mean r̄, 0.62 ± 0.19) than contralateral stimulation (mean r̄, 0.34 ± 0.30) (Fig. 12b, LME: r̄ ~ ctACS placement, [*F*_1, 13.8_ = 24.0, *p* < .001]). The response pattern, however, differed significantly. Ipsilateral stimulation resulted in a bimodal response in 7/8 CN cells whereas for contralateral stimulation only 1/8 showed a bimodal response.

**Figure 13:**
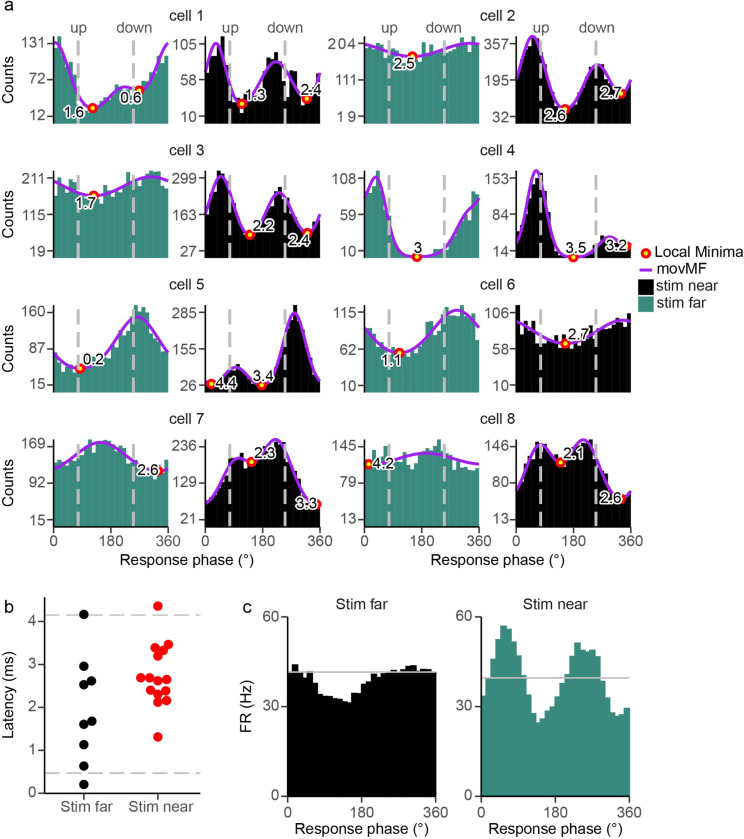
Near and far ctACS electrode locations recruit different proportions of PC populations. **(a)** At 80 Hz, histograms of each CN cell’s entrainment in response to both ctACS locations: one is adjacent to the recording site (Stim near, black), and location two is on the contralateral skull surface (Stim far, green). Red circles filled with yellow show the latency of the CN minima from the peaks of the up or down PC populations (weighed mean vector, gray line). **(b)** The distribution of the latencies in response to both ctACS locations at 80 Hz. Gray horizontal lines represent two SD from the mean. **(c)** Histograms show the entrainment of near and far ctACS location for 80 Hz, n=8. The gray horizontal line is the average spontaneous firing rate at each ctACS location.

We further explored whether these ctACS locations recruit different proportions of the PC population, up or down PCs. As was done for the entire CN population earlier (Fig. 11a), we calculated the latencies of the troughs in CN activity relative to the population vectors of the up and down PCs, for each ctACS location (ipsilateral and contralateral) for 80 Hz (Fig. 13a). For the contralateral stimulation group, we found that 6/8 cells had latencies calculated from the up-PC population and 2/8 from the down-PC population. For the ipsilateral stimulation group, 7/8 cells were bimodal, and so had latencies calculated from PC populations. The latencies for the ipsilateral and contralateral stimulation were similar (Fig. 13b, LMER model: (Latency ~ stim placement [*F*_1,16.7_ = 3.92, *p* = 0.064]). The similar latency is consistent with the modulation of the CN with contralateral stimulation being due to modulation of the same PCs activated with ipsilateral stimulation. The unimodal nature of the response to contralateral stimulation and the CN trough following the up PC peak might mean that at more distant sites from the stimulation electrode the local E-field direction is in phase and directed inward from the local pial surface, resulting in all PCs modulating in phase with respect to each other and the AC stimulus. Population histograms show the collective CN entrainment pattern of each ctACS location (Fig. 13b). Of note, the contralateral stimulation resulted in a weaker unimodal distribution whereas, similar to the whole CN population, these CN cells had a strong bimodal response to ipsilateral stimulation.

In sum, the strength and pattern of the entrainment depends on the stimulus electrode location: the closer the AC stimulation is to the CN population of interest, the stronger the entrainment and the more likely it will induce a bimodal response.

## Discussion

Here, we investigated how ctACS entrains PC and Lat CN cells *in vivo*. PC and Lat CN cells were found to exhibit differential entrainment patterns at the individual and population levels that varied in a frequency-dependent manner. Both cell types showed increasingly strong modulation with increasing stimulus frequency. Individual PCs predominantly exhibited unimodal responses to ctACS, but at higher frequencies (50 and 80 Hz), the population response was bimodal, with two groups of PCs responding ~ 180° out of phase to each other. In contrast, most CN cells displayed unimodal responses at lower frequencies but had bimodal response patterns at higher stimulus frequencies. As a population, the CN response was also bimodal. Analysis of the phase relationship between PC and CN activity across stimulus frequencies supported the hypothesis that ctACS modulates CN activity indirectly as a result of modulating PC activity. Some of these results align with those found in prior studies using surface stimulation of the cerebellum, whereas others differ, as discussed below.

### PC responses to cortical and transcranial stimulation

Our prior work demonstrated that AC stimulation applied directly to the cerebellar cortex strongly entrains PC and CN cells, raising the possibility that ctACS could be used to modulate cerebellar output to influence brainstem and forebrain activity [15, 16, 26]. These studies provided information on the CN and PC modulation patterns during AC stimulation, the non-radial nature of the induced E-field in the cerebellum, and evidence for direct polarization of PC and CN cells [15, 16, 26]. However, in ctACS the electrical current is applied outside of the skull, which will alter the resulting E-field within the cerebellum. In particular, compared to direct cerebellar AC stimulation, the E-field generated by ctACS is weaker and likely has a different distribution. Indeed, comparison of brain surface and transcranial stimulation of the cerebrum showed that the latter generates a much weaker E-field within the brain [19], which is also the case with ctACS and the cerebellum (data not shown). Thus, it was worth investigating whether the response of cerebellar neurons, particularly that of CN neurons, to ctACS differed significantly from what was found with direct cerebellar stimulation.

With ctACS, the entrainment patterns of most PCs were unimodal and frequency-dependent, largely matching the prior direct cerebellar stimulation results [15, 26]. Also consistent with direct cerebellar stimulation results, the PC population response was bimodal, with the activity of the two sets of PCs being ~ 180° out of phase (up and down PCs), suggesting that two distinct PC populations are being recruited. The 180° phase relationship of these PC populations reflect differences in PC dendritic orientation relative to that of the local E-field in the vicinity of the PC. Several factors may determine the E-field orientation, including inhomogeneities in the brain resistance and the folding of the cerebellar cortex. In our prior study, the direction of the local E-field was found to vary sharply within the cerebellum, supporting the importance of inhomogeneous resistance as an important factor [15]. Regardless of the specific mechanism, the current results indicate that the PC population response to ctACS is likely to be bimodal (i.e., twice the stimulus frequency) at least for frequencies in the 50–80 Hz range. Thus, CN cells would often experience a modulated synaptic input at twice the stimulation frequency.

One important difference between direct cerebellar stimulation and ctACS was the phases of the circular mean vectors of the PC population. With direct stimulation of the cerebellar surface, PC vectors were clustered around phases of 90° or 270° relative to the AC cycle, and did not systematically change with stimulus frequency [15]. This is consistent with their modulation resulting from a direct effect of the E-field on PC membrane potential, where one would expect the maximal firing rates to coincide with the peaks of the AC stimulation [15]. In contrast, with ctACS, the phases of the circular mean vectors were somewhat earlier than 90° and 270° for 50 Hz stimulation, and the vectors showed a small (not-statistically significant) counterclockwise rotation in their phases going from 50 to 80 Hz stimulation. While we don’t have a definitive explanation for the phase advance at 50 Hz, one possibility is that with brain surface stimulation direct polarization of the PC is the dominant effect of the E-field on PCs, whereas with ctACS, the PC response modulation of molecular layer interneurons (MLIs) may contribute to a greater degree (see later section on mechanisms). This could cause the phase of the circular mean vector to shift earlier. Moreover, while the counterclockwise rotation of the population vectors between 50 Hz and 80 was not statistically significant, the direction of the rotation was as expected if part of the response was due to modulation of synaptic input, and the small magnitude of the rotation could reflect both the short conduction time of action potentials of the interneurons to the PCs and that the response is a mix of direct and synaptic modulation.

### CN responses to direct and transcranial cerebellar stimulation.

Two prior studies from our laboratories investigated CN responses to directional AC stimulation of the cerebellar surface. Avlar et al. (2023) tested frequencies between 0.5 Hz and 20 Hz (but primarily between 0.5 Hz and 5 Hz) and found that most CN cells showed an unimodal response and only a minority showed a bimodal one. Moreover, the amplitude of the modulation did not significantly change over that range (a small, non-significant decrease was reported). In contrast, Kang et al. (Submitted) tested a higher range of frequencies (40 Hz − 400 Hz) with lower stimulus intensities (that would be less likely to cause a direct modulation of CN cells) and showed that nearly half (42%) of the population showed bimodal responses and that modulation strength increased significantly as the stimulus frequency was increased to peak around 100 Hz before decreasing. The differences in modulation observed in these two studies could reflect the different frequency ranges that were tested and/or the stimulus intensities used.

The present results help reconcile these apparent differences in CN modulation and indicate that the differences are largely due to the different frequency ranges that were tested. Here, the tested frequencies overlapped with those used in both prior studies, and we found that modulation strength was relatively flat at lower frequencies (actually dipped slightly between 0.5 Hz and 5 Hz) before rising significantly to peak around 80 Hz, matching the results of both prior studies. Moreover, we observed a rise in the proportion of bimodal cells as the stimulus frequency increased, potentially explaining the greater prevalence found in the Kang et al study.

One difference between the direct and ctACS results, however, is that a much higher proportion of bimodal cells at the higher stimulus frequencies was found with ctACS: brain surface, 42% ([27] versus ctACS, 91% at 80 Hz. This may be due to differences in the distribution of PCs that would be activated by direct surface versus transcranial stimulation, which we propose is the mechanism underlying the bimodal CN responses (see next section).

One question that arises is whether the differing CN responses to ctACS (uni- versus bi-modal) reflect the responses of distinct CN cell types, particularly given the increasing number of CN cell classes that can be defined based on morphological and physiological properties [29]. While it is likely that different CN cell types will show distinct responses to ctACS, cell type cannot fully underlie the bimodal and unimodal responses, as individual CN cells showed both patterns, typically transitioning from unimodal to bimodal as stimulus frequency was increased. However, while this transition occurred between 20 Hz and 50 Hz for most cells, some transitioned at higher or lower frequencies, and this variation might reflect distinct CN cell types. Most intriguing in this regard is the subset of cells that showed a bimodal response to low (0.5 Hz) stimulation. Conclusive testing of this possibility will require recording from identified CN neurons.

### Mechanisms of entrainment.

The E-field induced by the AC stimulation can potentially influence neuronal firing by directly polarizing the cell’s membrane and by influencing the synaptic input to the cell. In trying to explain the responses of PCs and CN cells, we consider both direct polarization and synaptic effects.

Under *in vitro* conditions, PCs are activated with lower currents than MLIs when currents are applied directly to the cerebellar surface, most likely because of their larger dendritic trees with somatodendritic axes that are oriented parallel to the uniform E-field [30]. The situation is more complex under *in vivo* conditions, as the E-field is not uniform and not always directed inward from the pial surface [15], as was the case with the *in vitro* preparation. *In vivo*, with surface stimulation, the phases of the PC circular mean vectors tended to align with the peaks of the AC current (i.e., at 90° and 270°) across stimulus frequencies, consistent with a direct polarization of their cell membrane by the E-field being the predominant factor in the modulation of their activity.

As mentioned earlier, this was not the case with ctACS, where the phases of the PC circular mean vectors were not directed at 90° or 270° at 50 Hz stimulation, and showed a small, albeit not significant, counterclockwise rotation for 80 Hz stimulation. To explain this phase shift, we suggest that both direct membrane polarization and synaptically mediated effects contribute to the PC response to ctACS. Both MLIs and granule cells provide significant synaptic input to PCs. MLIs are inhibitory, and at least those in the lower parts of the molecular layer largely modulate in the same phase as PCs [30]. Thus, their activity would act on PC activity at a short delay (due to the axonal conduction time and the synaptic delay), and as a result, would preferentially inhibit the lagging tail of the PC response, which would result in a clockwise shift of the phase of the circular mean vector from 90° or 180°, as was observed.

Granule cells provide the vast majority of synaptic input to PCs and modulation of their activity could significantly impact PC responses to ctACS; however, it seems unlikely that modulation of their activity plays a significant role for several reasons. They have just a few short dendrites and their somata are roughly centered within the 4–6 radially extending dendrites, characteristics that would favor weak to little modulation of their activity by an external E-field [30, 31]. Polarization of presynaptic terminals can influence transmitter release [32, 33]; however, the axons of the granule cells form mostly en passant type synapses, which are unlikely to be significantly polarized, as the ends of neuronal processes are the primary sites of polarization by an imposed E-field [32]. Anatomical estimates put the number of synapses made by a single parallel fiber at somewhat less than 1,000 (675, [34] and ~ 780 based on assuming a parallel fiber length of ~ 5 mm and the data given in [35]. Thus, only about 0.1–0.2% of the parallel fiber synapses onto PCs are end terminal type synapses that would potentially be subject to strong polarization. One caveat is that activation of an even smaller fraction of the total synapses onto PCs is sufficient to elicit spikes [36], so it is possible that modulation of parallel fiber synapses could make a small contribute to the overall modulation of PC by ctACS. Finally, we note that Golgi cells have a large apical dendritic tree in the same general orientation as PCs (i.e., extending superficially from the soma toward the pial surface), and therefore might be strongly modulated by the E-field in phase with PCs. This modulation could then modulate granule cell activity in a way that could act synergistically with the MLIs (depending on the time lag of the parallel fiber conduction). In sum, it seems plausible that PC responses to ctACS seem to be at least partially dependent on modulation of their afferents, particularly from the MLIs, in addition to direct polarization of their cell membrane.

As with PCs, we consider direct polarization of CN cells and modulation of their afferents as mechanisms by which ctACS modulated CN activity. With cerebellar surface stimulation, the response of CN neurons was consistent with a direct polarization being the dominant factor [16]. In particular, most CN cells had circular mean vectors with phases close to 90° or 270° at most stimulus frequencies (note, however, that only relatively low frequencies were tested for most cells where the phase shift reflecting the afferent conduction time would be relatively small and potentially hard to detect), and the magnitude of the E-field at CN depths was large enough to be suprathreshold for driving spikes [15]. In contrast, with ctACS at the intensities used here, the E-field amplitude at CN depths was significantly smaller (~ 0.05–0.10 mV/mm; data not shown). These values are significantly lower than the threshold for stimulation of the cerebral cortical neurons (pyramidal cells > 28 mV/mm and interneurons ~ 44 mV/mm; [37], and thus, likely below threshold for CN neurons. However, weak E-fields (~ 0.05–0.10 mV/mm), similar to what ctACS generated here, produce a sufficient subthreshold polarization of these cell membrane potential to modulate the spike activity [37]. So, it is possible that direct polarization of CN cells may contribute to the responses we observed.

Nevertheless, the results strongly suggest that the dominant factor is modulated synaptic input due to the modulation of PC activity. The variation in the strength of the modulation (r̄) of the PCs and CN cells with stimulus frequency was highly correlated. Furthermore, the bimodal response of PCs on a population level, particularly to the higher stimulus frequencies provides a pattern of activity that could underlie the bimodal responses of individual CN cells, and the increasing prevalence of bimodal CN responses at these higher frequencies is parallelled by the population level responses of PCs. Thus, unimodal CN responses occur when a CN cell receives inputs from PCs that all have circular mean vectors with similar phases, and bimodal responses are due to a CN cell receiving input from two sets of PCs with vectors that are ~ 180° apart. A key question is whether PC modulation is appropriately timed to produce the observed CN modulation. Given that PCs are inhibitory, their activity would reduce CN activity at a latency approximately equal to the conduction time of the PC axon to the CN. Here, we showed that this is the case in that the local minima of individual CN cells matched the times when the peak inhibitory synaptic input from a PC should have occurred, given the conduction time of the PC axon to the CN.

### Translational relevance

It is not clear how ctACS produces its therapeutic effects, but likely its actions are at least partly mediated by affecting activity and plasticity in cerebellar target regions, particularly in the forebrain. This underscores the importance of understanding how the CN, the major output station of the cerebellum, responds to ctACS, something that has not been previously studied at a cellular level. Here, we elucidated the response pattern of CN cells to ctACS, highlighting the parameters for driving Lat CN cells that are likely translatable and could help guide clinical trials. Our results suggest that this should be done for future cognitive clinical studies. In particular, our results indicate that stimulus frequency is a critical parameter. The modulation strength (r̄) increased across frequency, plateauing around 80 Hz for PC and CN cells. Intriguingly, a collection of studies involving ctACS using mid-high gamma‐band (40–85 Hz) frequencies was found to improve motor learning [11]. Moreover, the response pattern (unimodal versus bimodal) is frequency dependent, and the cerebellar output is actually modulated at twice the stimulus frequency in bimodal cells. The importance of this is highlighted by neuromodulation studies of forebrain structures that show efficacy is highly dependent on stimulus frequency [38]. Thus, if ctACS is used to target forebrain areas, the stimulus frequency doubling of the CN output should be taken into account.

Another critical variable is the placement of ctACS electrode relative to the cell population of interest. Our data showed that different electrode placements may recruit a different proportion of the PC population. The ability of ctACS to activate different PC populations based on electrode placement would allow modulation of distinct CN cell populations; this is important since CN regions project to different forebrain regions involved in distinct cognitive and motor function.

## Conclusion

In conclusion, our study demonstrates that transcranial electrical stimulation induces a frequency-dependent modulation of PC and CN activity. We observed predominantly unimodal responses to ctACS stimulation by individual PCs. However, the response was bimodal at the population level. In contrast, most CN cells exhibited bimodal response patterns, especially at higher stimulus frequencies. Our findings support the hypothesis that ctACS primarily modulates cerebellar output by modulating PC activity, which in turn drives CN activity. These results may guide future studies on the impact of ctACS on animal models or human patients with motor and cognitive impairments, potentially leading to new frameworks for understanding neurological disorders and treatments.

## Data Availability

The scientific data will be made freely available for download from a publicly accessible repository, such as Dryad, in compliance with NIH policies.
